# Traditional medicine and popular remedies in mental health care within an Arab-Muslim context: complementarity and challenges

**DOI:** 10.1192/j.eurpsy.2026.11448

**Published:** 2026-07-31

**Authors:** F. Zaouali, I. Anes

**Affiliations:** 1 Emergency department and mobile emergency and resuscitation service, Tahar Sfar University Hospital, Mahdia; 2 Outpatient psychiatric consultation, Hadj Ali Soua Regional Hospital, Ksar Hellal, Monastir, Tunisia

## Abstract

**Introduction:**

In many Arab-Muslim societies, traditional medicine and popular remedies are frequently utilized by individuals experiencing mental health issues. These practices coexist with modern psychiatric treatments, influencing patients’ pathways to care. Understanding the extent, motivations, and consequences of such practices is essential for culturally sensitive and effective mental health interventions.

**Objectives:**

To evaluate the prevalence, types, and perceived effectiveness of traditional and popular remedies used by psychiatric patients in an Arab-Muslim context, and to identify potential benefits and risks associated with these practices.

**Methods:**

A mixed-methods study was conducted in the the psychiatric outpatient consultation at hadj Ali Soua regional hospital over one year. Quantitative data were collected through structured questionnaires administered to 75 patients diagnosed with common mental disorders. Qualitative data were obtained via semi-structured interviews with 20 patients and 5 traditional healers. The questionnaire addressed types of traditional treatments used (herbal remedies, spiritual healing, amulets, religious rituals), motivations for use, perceived outcomes, and interaction with prescribed psychiatric treatments. Literature review focused on studies from Middle Eastern and North African contexts published between 2000 and 2024.

**Results:**

Seventy-two percent of patients reported using at least one form of traditional or popular remedy alongside psychiatric treatment. Herbal remedies (56%) and spiritual healing practices (48%) were the most common. Patients cited cultural beliefs, accessibility, and trust in traditional healers as primary motivations. While 60% perceived these remedies as beneficial for symptom relief and emotional support, 25% reported delays in seeking psychiatric care due to reliance on traditional methods. Interviews revealed mixed attitudes among traditional healers regarding integration with psychiatric services. Literature review corroborated these findings, highlighting both potential complementarities and risks such as treatment discontinuation and adverse interactions.

**Conclusions:**

Traditional medicine remains deeply embedded in mental health care among Arab-Muslim populations, offering psychosocial benefits but also posing challenges for clinical management. Culturally sensitive collaboration between psychiatrists and traditional healers may improve patient outcomes and treatment adherence. Further research is needed to develop integrative care models and educational programs addressing this interface.

**Disclosure of Interest:**

None Declared

